# Supplementary material for: Personalised Risk Prediction in Hereditary Breast and Ovarian Cancer: A Protocol for a Multi-Centre Randomised Controlled Trial
Source: Cancers (Basel). 2022 May 31;14(11):2716. doi: 10.3390/cancers14112716 (PMC9179465; doi:10.3390/cancers14112716)
Supplement: Supplementary file 1 [file cancers-14-02716-s001.zip › S1.pdf]

# PRECISION-HBOC

**Study Title**: Stratifying Risk for Early Detection in Hereditary Breast and Ovarian Cancer

Baseline Questionnaire

**Investigators**: Professor Marc Tischkowitz, Professor Gareth Evans, Professor Allison Kurian, Dr Stephanie Archer, Antonis Antoniou, Nichola Fennell, Ellen Colvin, Rozelle Laquindanum and Meredith Mills.

Baseline Questionnaire (v.2.0: 18.11.2021) IRAS Project ID: 291629 R&D Number: A095733

—-------------------------------------------------------------------

We would be grateful if you could answer the following questions about yourself and your health.

The questions in this survey aim to explore your knowledge and perceptions of breast cancer risk and your current mental/physical well-being. Some questions will also ask you about whether you have used any NHS services in the last 6 months.

Please try to answer every question as completely and honestly as you can - your answers will be treated as strictly confidential and will only be used for medical research.

Contact details for sources of further information and support are provided at the end of this questionnaire.

All information will be treated as strictly confidential.

This survey should take between 15 and 20 minutes to complete.

Thank you for completing this questionnaire.

3. Please enter your participant ID - this is found on the letter included in your information pack.

____________________________________________________

**4. Which of the following numbers do you think represents the biggest risk of getting a disease?**

a) 1 in 100
b) 1 in 1000
c) 1 in 10

**5.**

| | Not at all like me (1) | A little bit like me (2) | Somewhat like me (3) | Very like me (4) | Entirely like me (5) |
|---|---|---|---|---|---|
| Unforeseen events upset me greatly (1) | o | o | o | o | o |
| It frustrates me not having all the information I need (2) | o | o | o | o | o |
| Uncertainty keeps me from living a full life (3) | o | o | o | o | o |
| One should always look ahead so as to avoid surprises (4) | o | o | o | o | o |
| A small unforeseen event can spoil everything, even with the best of planning (5) | o | o | o | o | o |

| | | | | | |
|---|---|---|---|---|---|
| When it's time to act, uncertainty paralyses me (6) | o | o | o | o | o |
| When I am uncertain I can't function very well (7) | o | o | o | o | o |
| I always want to know what the future has in store for me (8) | o | o | o | o | o |
| I can't stand being taken by surprise (9) | o | o | o | o | o |
| The smallest doubt can stop me from acting (10) | o | o | o | o | o |
| I should be able to organize everything in advance (11) | o | o | o | o | o |
| I must get away from all uncertain situations (12) | o | o | o | o | o |

**6. How likely are you to get breast cancer in the next 10 years?**

a) Very unlikely
b) Unlikely
c) Neither likely nor unlikely
d) Likely
e) Very likely

**7. On a scale from 0 to 100, where 0 = certain not to happen, and 100 = certain to happen, how likely do you think you are to get breast cancer in the next 10 years?**

| | | | | | | | | | | |
|---|---|---|---|---|---|---|---|---|---|---|
| **0** | **10** | **20** | **30** | **40** | **50** | **60** | **70** | **80** | **90** | **10 0** |

**Move slider to answer ()**

**8. How likely are you to get breast cancer in your lifetime?**

a) Very unlikely
b) Unlikely
c) Neither likely nor unlikely
d) Likely; and
e) Very likely

**9. On a scale from 0 to 100, where 0 = certain not to happen, and 100 = certain to happen, how likely do you think you are to get breast cancer in your lifetime?**

| | | | | | | | | | | |
|---|---|---|---|---|---|---|---|---|---|---|
| **0** | **10** | **20** | **30** | **40** | **50** | **60** | **70** | **80** | **90** | **10 0** |

**Move slider to answer ()**

**10. Compared to other women your age and ethnicity, how likely are you to get breast cancer in the next 10 years?**

a) Much below average
b) Below average
c) Same average risk as other women your age
d) Above average
e) Much above average

**11. Compared to other women your age and ethnicity , how likely are you to get breast cancer in your lifetime?**

a) Much below average
b) Below average
c) Same average risk as other women your age
d) Above average
e) Much above average

**12. How worried are you about getting breast cancer in the next 10 years?**

a) Not at all worried
b) Slightly worried
c) Somewhat worried
d) Worried
e) Very worried

**13. How worried are you about getting breast cancer in your lifetime?**

a) Not at all worried
b) Slightly worried
c) Somewhat worried
d) Worried
e) Very worried

**14. During the past month, how often have you thought about your chances of developing breast cancer?**

a) Not at all
b) Rarely
c) Sometimes

d) Often
e) Almost all the time

**15. During the past month, how often have your thoughts about your chances of getting breast cancer affected your mood?**

a) Not at all
b) A little
c) Somewhat
d) A lot

**16. During the past month, how often have your thoughts about your chances of getting breast cancer affected your ability to perform your daily activities?**

a) Not at all
b) A little
c) Somewhat
d) A lot

**17.** *A number of statements which people have used to describe themselves are given below. Read each statement and then select the appropriate answer to indicate how you feel right now, that is, at this moment. There are no right or wrong answers. Do not spend too much time on any one statement but give the answer which seems to describe your present feelings best.*

| | *Not at all (1)* | *Somewhat (2)* | *Moderate (3)* | *Very much so (4)* |
|---|---|---|---|---|
| *I feel calm (1)* | *o* | *o* | *o* | *o* |
| *I am tense (2)* | *o* | *o* | *o* | *o* |
| *I feel upset (3)* | *o* | *o* | *o* | *o* |

| | | | | |
|---|---|---|---|---|
| *I am relaxed (4)* | o | o | o | o |
| *I feel content (5)* | o | o | o | o |
| *I am worried (6)* | o | o | o | o |

**18.** *The following questions ask for your views about your health, how you feel and how well you are able to do your usual activities. If you are unsure about how to answer any questions, please give the best answer you can. Do not spend too much time in answering as your immediate response is likely to be the most accurate.*

**19. In general, would your say your health is:**

a) Excellent
b) Very good
c) Good
d) Fair
e) Poor

*20. The following questions are about activities you might do during a typical day:*

**21. Does your health limit you in these activities? If so, how much?**

Moderate activities, such as moving a table, pushing a vacuum, bowling or playing golf

a) Yes, limited a lot
b) Yes, limited a little
c) No, not limited at all

22. Climbing several flights of stairs

a) Yes, limited a lot
b) Yes, limited a little
c) No, not limited at all

**23. During the past 4 weeks, have you had any of the following problems with your work or other regular daily activities as a result of your physical health:**

Accomplished less than you would like

a) Yes
b) No

24. Were limited in the kind of work or other activities

a) Yes
b) No

**25. During the past 4 weeks, have you had any of the following problems with your work or other regular daily activities as a result of any emotional problems (such as feeling depressed or anxious?):**

Accomplished less than you would like

a) Yes
b) No

26. Didn't do work or other activities as carefully as usual

a) Yes
b) No

**27. During the last 4 weeks, how much did pain interfere with your normal work (including work outside of the home and housework?)**

a) Not at all
b) A little bit
c) Moderately
d) Quite a bit
e) Extremely

**28.** *These questions are about how you feel and how things have been with you during the last month. For each question, please indicate the one answer that comes closest to the way you have been feeling.*

**How much time during the last month:**

| | All of the time (1) | Most of the time (2) | A good bit of the time (3) | Some of the time (4) | A little of the time (5) | None of the time (6) |
|---|---|---|---|---|---|---|
| **Have you felt calm and peaceful? (1)** | o | o | o | o | o | o |
| **Did you have a lot of energy? (2)** | o | o | o | o | o | o |
| **Have you felt downhearted and low? (3)** | o | o | o | o | o | o |
| **Has your health limited your social activities (like visiting friends or close relatives)? (4)** | o | o | o | o | o | o |

29. This section will ask about hospital and GP visits you have had in the last 6 months

30. **Have you used any of the following services in the last 6 months? If yes, how many times?**

| | Have you used this service? | | | How many times did you use this service? |
|---|---|---|---|---|
| | Yes (1) | No (2) | Don't Know (3) | Number of uses (1) |
| GP (at a surgery/practice) (1) | o | o | o | |
| GP (at your home) (2) | o | o | o | |

| | | | | |
|---|---|---|---|---|
| **Practice Nurse (at the surgery) (3)** | o | o | o | |
| **Nurse (at your home) (4)** | o | o | o | |
| **Walk in centre (5)** | o | o | o | |

31. If you have been to the GP or practice, please tell us why below. Please tell us about each visit separately.

________________________________

**32. Have you attended Accident and Emergency (A&E) in the last 6 months?**

o Yes (1) o No (2) o Don't know (3)

**Display This Question:**

**If Have you attended Accident and Emergency (A&E) in the last 6 months? = Yes**

**Q33 How many times did you attend Accident and Emergency (A&E) in the last 6 months that DID NOT result in a hospital admission?**

________________________________________________________________

**Display This Question:**

**If Have you attended Accident and Emergency (A&E) in the last 6 months? = Yes**

**Q34 How many times did you attend Accident and Emergency (A&E) in the last 6 months that DID result in you being admitted into hospital as an inpatient?**

________________________________________________________________

**Q35 Please tell us whether you attended the hospital in the last 6 months for any of the below appointments, which departments you attended and how many times you attended.**

| | Did you attend? | How many times? | Which departments did you attend? How many times for each? |
|---|---|---|---|

| | Yes (1) | No (2) | Don't Know (3) | Number (1) | Department (1) |
|---|---|---|---|---|---|
| **Planned hospital outpatient appointments (4 hours or less) (1)** | o | o | o | | |
| **Planned hospital outpatient appointments (more than 4 hours but NOT overnight) (2)** | o | o | o | | |

| | | | | | |
|---|---|---|---|---|---|
| **Planned hospital overnight stays (3)** | o | o | o | | |

**Q36 Have you used any community based health/social care services in the last 6 months? (e.g. social worker, home help, care worker, occupational therapy or physiotherapy).**

**Please tell us what services you used (if any), and how many times you used them.**

________________________________________________________________

Q37 Are you currently taking any regular medicines? (Prescribed by a GP/hospital doctor or over-the-counter medications)

o Yes (1) o No (2) o Don't know (3)

Display This Question:

If Are you currently taking any regular medicines? (Prescribed by a GP/hospital doctor or over-the-c... = Yes

Q38 Please give some further details of the medications you are currently taking. (Names, how long you've been taking them, how often you take them)

________________________________________________________________

Q39 These questions will ask you about your thoughts and feelings about risk management options if you are found to have inherited the disease-causing gene change (mutation) identified in your family.

Q40 Are you aware of the options available to women with a pathogenic gene change associated with an increased risk of certain types of cancer, such as the one in your family?

o Yes, very well informed (1)

o Kind of informed (2)

o No, I have no idea of options are available (3)

Q41 If yes, how have you accessed this information?

________________________________________________________________

Q42 What risk management options do you think are available for women found to carry a disease-causing gene change (mutation), such as the one in your family? (Select as many as you like)
(NB: some of these options are common misconceptions of risk management, and are not valid ways of managing risk)

▢ Becoming a vegetarian (1)

▢ Risk reducing breast surgery (double mastectomy) (2)

▢ Gene therapy (3)

▢ Risk reducing ovarian surgery (surgery to remove the ovaries and fallopian tubes) (4)

▢ Ovarian screening (5)

- ▢ Chemoprevention (medication to reduce your risk of cancer) (6)
- ▢ Annual breast screening (7)
- ▢ Regular smear tests (8)
- ▢ Stopping smoking (9)
- ▢ Avoiding underwire bras (10)
- ▢ Hysterectomy ( surgery to remove the womb) (11)
- ▢ Paracetamol (12)

Q43 Have you thought about what risk management options you would like to take, if you inherited the gene change?

o Yes I have thought about it a lot (1)

o I have thought about it a little (2)

o No, I have not thought about it at all (3)

Q44 Have you considered taking any of the below surgical options to manage breast/ovarian cancer risk?

| | Have you thought about this option? | | Would you consider taking this option? | | | If yes, when would you consider taking this option? | | | | |
|---|---|---|---|---|---|---|---|---|---|---|
| | Yes (1) | No (2) | Yes (1) | No (2) | Don't Know (3) | I have already done this (1) | As soon as possible. I am seeking an appointment, or have one booked. (2) | In the next 6 months (3) | In the future, but not in the next 6 months (4) | I'm not sure (5) |

| | | | | | | | | | | |
|---|---|---|---|---|---|---|---|---|---|---|
| Risk Reducing Breast Surgery (double mastectomy) (2) | o | o | o | o | o | o | o | o | o | o |
| Risk Reducing Ovarian Surgery (bilateral salpingo-oophorectomy) (3) | o | o | o | o | o | o | o | o | o | o |

Q45 Have you considered the option of annual breast screening to manage breast cancer risk?

| | Have you thought about this option? | | Would you consider taking this option? | | | If yes, when would you consider taking this option? | | | | | |
|---|---|---|---|---|---|---|---|---|---|---|---|
| | Yes (1) | No (2) | Yes (1) | No (2) | Don't Know (3) | I have already done this and plan on continuing screening (1) | I have already done this but would not do it | As soon as possible. I am seeking an appointment, or have one booked. (3) | In the next 6 months (4) | In the future, but not in the next 6 mon | I'm not sure (6) |

| | | | | | | | | | | |
|---|---|---|---|---|---|---|---|---|---|---|
| | | | | | | agai n (2) | | | ths (5) | |
| Annual Breast Screening (1) | o | o | o | o | o | o | o | o | o | o |

Q46 If you would consider any of the above options in the future (but not in the next 6 months), what are your considerations on the timing of this option? (e.g. want to wait until a certain age, don't feel ready yet, want to complete your family...)

________________________________________________________________

Q47 On the scale below, please tell us about how you feel about the option of annual breast screening to manage cancer risk?

| | 1 (1) | 2 (2) | 3 (3) | 4 (4) | 5 (5) | 6 (6) | 7 (7) | 8 (8) | 9 (9) | 10 (10) | |
|---|---|---|---|---|---|---|---|---|---|---|---|
| Beneficial | o | o | o | o | o | o | o | o | o | o | Harmful |
| Important | o | o | o | o | o | o | o | o | o | o | Unimportant |

| | | | | | | | | | | | |
|---|---|---|---|---|---|---|---|---|---|---|---|
| Good thing | o | o | o | o | o | o | o | o | o | o | Bad thing |
| Pleasant | o | o | o | o | o | o | o | o | o | o | Unpleasant |

Q48 On the below, please tell us about how you feel about the option of risk reducing breast surgery to manage cancer risk?

| | 1 (1) | 2 (2) | 3 (3) | 4 (4) | 5 (5) | 6 (6) | 7 (7) | 8 (8) | 9 (9) | 10 (10) | |
|---|---|---|---|---|---|---|---|---|---|---|---|
| Beneficial | o | o | o | o | o | o | o | o | o | o | Harmful |
| Important | o | o | o | o | o | o | o | o | o | o | Unimportant |
| Good thing | o | o | o | o | o | o | o | o | o | o | Bad thing |
| Pleasant | o | o | o | o | o | o | o | o | o | o | Unpleasant |

Q49 On the below, please tell us about how you feel about the option of risk reducing ovarian surgery to manage cancer risk?

| | 1 (1) | 2 (2) | 3 (3) | 4 (4) | 5 (5) | 6 (6) | 7 (7) | 8 (8) | 9 (9) | 10 (10) | |
|---|---|---|---|---|---|---|---|---|---|---|---|
| Beneficial | o | o | o | o | o | o | o | o | o | o | Harmful |
| Important | o | o | o | o | o | o | o | o | o | o | Unimportant |
| Good thing | o | o | o | o | o | o | o | o | o | o | Bad thing |
| Pleasant | o | o | o | o | o | o | o | o | o | o | Unpleasant |

Q50 What factors or influences have contributed to your answer above about risk management options?

________________________________________________________________

Q51 This final section asks for some personal details that are used in the personalised risk prediction tool, CanRisk, that will be used in this study.

Q52 How tall are you? (Please specify unit of measurement)

________________________________________________________________

Q53 What is your current weight? (Please specify unit of measurement)

_______________________________________________________________

Q54 Do you drink alcohol? If so, how many units on average do you drink?

| | How many units? | How often? | | |
|---|---|---|---|---|
| | Number (1) | Daily (1) | Weekly (2) | Monthly (3) |
| Glass of Wine (175 ml) (13) | | o | o | o |

| | | | | |
|---|---|---|---|---|
| Pint of Beer / Lager / Cider (568 ml) (14) | | o | o | o |
| Bottle of Beer (Standard 330 ml) (15) | | o | o | o |
| Alcoholic Pop Drink (275 ml) (16) | | o | o | o |
| Shot of spirits (25 ml) includes gin, rum, vodka, whisky, tequila, sambuca (17) | | o | o | o |

Q55 How old were you when you had your first period? (If above 18 or below 8 enter closest age)

| | 8 9 10 11 12 13 14 15 16 17 18 |
|---|---|
| Age (years) () | |

Q56 Have you ever taken the Oral Contraceptive Pill?

o Yes (1) o No (2)

Display This Question:

If Have you ever taken the Oral Contraceptive Pill? = Yes

Q57 How many years in total did you take the Oral Contraceptive Pill?

o Less than 1 year (4)

o 1-4 years (5)

o 5-9 years (6)

o 10-14 years (7)

o 15 or more years (8)

Display This Question:

If Have you ever taken the Oral Contraceptive Pill? = Yes

Q58 Have you taken the pill in the last two years?

o Yes (1)

o No (2)

Q59 Have your periods now stopped completely? (That is, have you now gone at least 6 months without having a period and you are not pregnant or using any form of hormonal contraception.)

o Yes (1) o No (2)

Q60 Have you ever used hormone replacement therapy (HRT) for the menopause?

o Yes (1) o No (2)

Q61 Have you ever had a mammogram?

o Yes (1)

o No (2)

Display This Question:

If Have you ever had a mammogram? = Yes

Q62 After a mammogram, some women may have been told about their breast density. Were you?

o Yes (1)

o No (2)

Display This Question:

If After a mammogram, some women may have been told about their breast density. Were you? = Yes

Q63 Was breast density measured using BI-RADS?

o Yes (1)

o No (2)

Display This Question:

If Was breast density measured using BI-RADS? = Yes

Q64 What was the result?

- o BI-RADS a (1)
- o BI-RADS b (2)
- o BI-RADS c (3)
- o BI-RADS d (4)
- o Unknown (5)

Q65 Have you ever been diagnosed with endometriosis?

o Yes (1) o No (2)

Q66 Have you had your tubes tied (tubal ligation)?

o Yes (1) o No (2)

We thank you for your time spent taking this survey, it is greatly appreciated. Your response has been recorded.

If you have any questions or concerns, please get in touch with us via the below contact details.

Cambridge - Contact us via email (cuh.precision-hboc@nhs.net), or phone, 01223 216446. Please note that this number will direct you to our main Clinical Genetics service, where you can leave a message and request for the study team to call you back.

Manchester - [Email & telephone number]

Stanford - Please direct questions to study coordinator Rozelle Laquindanum via email - rlaquind@stanford.edu, or by phone [telephone number]
