# Supplementary material for: Personalised Risk Prediction in Hereditary Breast and Ovarian Cancer: A Protocol for a Multi-Centre Randomised Controlled Trial
Source: Cancers (Basel). 2022 May 31;14(11):2716. doi: 10.3390/cancers14112716 (PMC9179465; doi:10.3390/cancers14112716)
Supplement: Supplementary file 1 [file cancers-14-02716-s001.zip › S2.pdf]

PRECISION-HBOC

Investigators: Professor Marc Tischkowitz, Professor Gareth Evans, Professor Allison Kurian, Dr Stephanie Archer, Antonis Antoniou, Nichola Fennell, Ellen Colvin, Rozelle Laquindanum and Meredith Mills.

Follow Up Questionnaire (v.2.0: 18.11.2021) IRAS Project ID: 291629 R&D Number: A095733

We would be grateful if you could answer the following questions about yourself and your health.

The questions in this survey aim to explore your knowledge and perceptions of breast cancer risk and your current mental/physical well-being. Some questions will also ask you about whether you have used any NHS services in the last 6 months.

Please try to answer every question as completely and honestly as you can - your answers will be treated as strictly confidential and will only be used for medical research.

Contact details for sources of further information and support are provided at the end of this questionnaire.

All information will be treated as strictly confidential.

This survey should take approximately 15 minutes to complete.

Thank you for completing this questionnaire.

1. How likely are you to get breast cancer in the next 10 years?

a) Very unlikely
b) Unlikely
c) Neither likely nor unlikely
d) Likely
e) Very likely

2. On a scale from 0 to 100, where 0 = certain not to happen, and 100 = certain to happen, how likely do you think you are to get breast cancer in the next 10 years?

	0	10	20	30	40	50	60	70	80	90	10<br>0

**Move slider to answer ()**	

3. How likely are you to get breast cancer in your lifetime?

a) Very unlikely
b) Unlikely
c) Neither likely nor unlikely
d) Likely; and
e) Very likely

4. On a scale from 0 to 100, where 0 = certain not to happen, and 100 = certain to happen, how likely do you think you are to get breast cancer in your lifetime?

	0	10	20	30	40	50	60	70	80	90	10<br>0

**Move slider to answer ()**	

5. Compared to other women your age and ethnicity, how likely are you to get breast cancer in the next 10 years?

a) Much below average
b) Below average
c) Same average risk as other women your age
d) Above average
e) Much above average

6. Compared to other women your age and ethnicity, how likely are you to get breast cancer in your lifetime?

a) Much below average
b) Below average
c) Same average risk as other women your age
d) Above average
e) Much above average

7. How worried are you about getting breast cancer in the next 10 years?

a) Not at all worried
b) Slightly worried
c) Somewhat worried
d) Worried
e) Very worried

8. How worried are you about getting breast cancer in your lifetime?

a) Not at all worried
b) Slightly worried
c) Somewhat worried
d) Worried
e) Very worried

9. During the past month, how often have you thought about your chances of developing breast cancer?

a) Not at all
b) Rarely
c) Sometimes
d) Often
e) Almost all the time

10. During the past month, how often have your thoughts about your chances of getting breast cancer affected your mood?

a) Not at all
b) A little
c) Somewhat
d) A lot

11. During the past month, how often have your thoughts about your chances of getting breast cancer affected your ability to perform your daily activities?

a) Not at all
b) A little
c) Somewhat
d) A lot

12. *A number of statements which people have used to describe themselves are given below. Read each statement and then select the appropriate answer to indicate how you feel right now, that is, at this moment. There are no right or wrong answers. Do not spend too much time on any one statement but give the answer which seems to describe you present feelings best.*

	Not at all (1)	*Somewhat (2)*	*Moderate (3)*	*Very much so (4)*
I feel calm (1)	o	o	o	o
I am tense (2)	o	o	o	o
I feel upset (3)	o	o	o	o
I am relaxed (4)	o	o	o	o
I feel content (5)	o	o	o	o
I am worried (6)	o	o	o	o

a)

13. *The following questions ask for your views about your health, how you feel and how well you are able to do your usual activities. If you are unsure about how to answer any questions, please give the*

best answer you can. Do not spend too much time in answering as your immediate response is likely to be the most accurate.

14. In general, would your say your health is:

a) Excellent
b) Very good
c) Good
d) Fair
e) Poor

The following questions are about activities you might do during a typical day:

15. Does your health limit you in these activities? If so, how much?

Moderate activities, such as moving a table, pushing a vacuum, bowling or playing golf

a) Yes, limited a lot
b) Yes, limited a little
c) No, not limited at all

Climbing several flights of stairs

a) Yes, limited a lot
b) Yes, limited a little
c) No, not limited at all

16**. During the past 4 weeks, have you had any of the following problems with your work or other regular daily activities as a result of your physical health:**

Accomplished less than you would like

a) Yes
b) No

Were limited in the kind of work or other activities

a) Yes
b) No

17**. During the past 4 weeks, have you had any of the following problems with your work or other regular daily activities as a result of any emotional problems (such as feeling depressed or anxious?):**

Accomplished less than you would like

a) Yes
b) No

Didn't do work or other activities as carefully as usual

a) Yes
b) No

18. **During the last 4 weeks, how much did pain interfere with your normal work (including work outside of the home and housework?)**

a) Not at all
b) A little bit
c) Moderately
d) Quite a bit
e) Extremely

19. *These questions are about how you feel and how things have been with you during the last month. For each question, please indicate the one answer that comes closest to the way you have been feeling.*

How much time during the last month:

	All of the time (1)	Most of the time (2)	A good bit of the time (3)	Some of the time (4)	A little of the time (5)	None of the time (6)
Have you felt calm and peaceful? (1)	o	o	o	o	o	o
Did you have a lot of energy? (2)	o	o	o	o	o	o
Have you felt downhearted and low? (3)	o	o	o	o	o	o
Has your health limited your social activities (like visiting friends or close relatives)? (4)	o	o	o	o	o	o

20. Please select the statement that best describes what your genetic result means for you current breast health:

1. I definitely do not have breast cancer
2. I am unlikely to have breast cancer
3. I am likely to have breast cancer
4. I have breast cancer
5. I am very unlikely to develop breast cancer
6. I am unlikely to develop breast cancer
7. I am likely to develop breast cancer
8. I am very likely to develop breast cancer
9. This result does not tell me anything about my future likelihood of breast cancer
10. I do not know what my test result means

21. Please tell us how much you have used any of the following services in the last 6 months. *If you have not used a service please enter '0'.*

	**Have you used this service?**			**How many times did you use this service?**
	**Yes (1)**	**No (2)**	**Don't Know (3)**	**Number of uses (1)**
**GP (at a surgery/practice) (1)**	o	o	o	

**GP (at your home) (2)**	o	o	o	
**Practice Nurse (at the surgery) (3)**	o	o	o	
**Nurse (at your home) (4)**	o	o	o	
**Walk in centre (5)**	o	o	o	

22. If you have been to the GP or practice, please tell us why below. Please tell us about each visit separately.

____

23. Have you used any community based health/social care services in the last 6 months? (e.g. social worker, home help, care worker, occupational therapy or physiotherapy).

Please tell us what services you used (if any), and how many times you used them.

____

24Have you attended Accident and Emergency (A&E) in the last 6 months?

o Yes (1) o No (2) o Don't know (3)

Display This Question:

If Have you attended Accident and Emergency (A&E) in the last 6 months? = Yes

25 How many times did you attend Accident and Emergency (A&E) in the last 6 months that DID NOT result in a hospital admission?

____

Display This Question:

If Have you attended Accident and Emergency (A&E) in the last 6 months? = Yes

26 How many times did you attend Accident and Emergency (A&E) in the last 6 months that DID result in you being admitted into hospital as an inpatient?

____

27 Please tell us whether you attended the hospital in the last 6 months for any of the below appointments, which departments you attended and how many times you attended.

	Did you attend?	How many times?	Which departments did you attend? How many times for each?

	Yes (1)	No (2)	Don't Know (3)	Number (1)	Department (1)
Planned hospital outpatient appointments (4 hours or less) (1)	o	o	o		
Planned hospital outpatient appointments (more than 4 hours but NOT overnight) (2)	o	o	o		
Planned hospital overnight stays (3)	o	o	o		

28 Are you currently taking any regular medicines? (Prescribed by a GP/hospital doctor or over-the-counter medications)

o Yes (1) o No (2) o Don't know (3)

Display This Question:

If Are you currently taking any regular medicines? (Prescribed by a GP/hospital doctor or over-the-c... = Yes

29 Please give some further details of the medications you are currently taking. (Names, how long you've been taking them, how often you take them)

____

These questions will ask you about your thoughts and feelings about risk management options if you are found to have inherited the disease-causing gene change (mutation) identified in your family.

Q30 Are you aware of the options available to women with a pathogenic gene change associated with an increased risk of certain types of cancer, such as the one in your family?

o Yes, very well informed (1)

o Kind of informed (2)

o No, I have no idea of options are available (3)

Q31 If yes, how have you accessed this information?

____

Q32 What risk management options do you think are available for women found to carry a disease-causing gene change (mutation), such as the one in your family? (Select as many as you like)
(NB: some of these options are common misconceptions of risk management, and are not valid ways of managing risk)

▢ Becoming a vegetarian (1)

▢ Risk reducing breast surgery (double mastectomy) (2)

▢ Gene therapy (3)

▢ Risk reducing ovarian surgery (surgery to remove the ovaries and fallopian tubes) (4)

▢ Ovarian screening (5)

▢ Chemoprevention (medication to reduce your risk of cancer) (6)

▢ Annual breast screening (7)

▢ Regular smear tests (8)

▢ Stopping smoking (9)

▢ Avoiding underwire bras (10)

▢ Hysterectomy (surgery to remove the womb) (11)

▢ Paracetamol (12)

Q33 Have you thought about what risk management options you would like to take, if you inherited the gene change?

o Yes I have thought about it a lot (1)

o I have thought about it a little (2)

o No, I have not thought about it at all (3)

Q34 Have you considered taking any of the below surgical options to manage breast/ovarian cancer risk?

	Have you thought about this option?		Would you consider taking this option?			If yes, when would you consider taking this option?				
	Yes (1)	No (2)	Yes (1)	No (2)	Don't Know (3)	I have already done this (1)	As soon as possible. I am seeking an appointment, or have one booked. (2)	In the next 6 months (3)	In the future, but not in the next 6 months (4)	I'm not sure (5)
Risk Reducing Breast Surgery (double	o	o	o	o	o	o	o	o	o	o

mastectomy) (2)										
Risk Reducing Ovarian Surgery (bilateral salpingo-oophorectomy) (3)	o	o	o	o	o	o	o	o	o	o

Q35 Have you considered the option of annual breast screening to manage breast cancer risk?

	Have you thought about this option?		Would you consider taking this option?			If yes, when would you consider taking this option?					
	Yes (1)	No (2)	Yes (1)	No (2)	Don't Know (3)	I have already done this and plan on continuing screening (1)	I have already done this but would not do it again (2)	As soon as possible. I am seeking an appointment, or have one booked. (3)	In the next 6 months (4)	In the future, but not in the next 6 months (5)	I'm not sure (6)
Annual Breast Screening (1)		o	o	o	o	o	o	o	o	o	o

Q36 If you would consider any of the above options in the future (but not in the next 6 months), what are your considerations on the timing of this option? (e.g. want to wait until a certain age, don't feel ready yet, want to complete your family...)

____

Q37 On the scale below, please tell us about how you feel about the option of annual breast screening to manage cancer risk?

	1 (1)	2 (2)	3 (3)	4 (4)	5 (5)	6 (6)	7 (7)	8 (8)	9 (9)	10 (10)	
Beneficial	o	o	o	o	o	o	o	o	o	o	Harmful
Important	o	o	o	o	o	o	o	o	o	o	Unimportant
Good thing	o	o	o	o	o	o	o	o	o	o	Bad thing
Pleasant	o	o	o	o	o	o	o	o	o	o	Unpleasant

Q38 On the below, please tell us about how you feel about the option of risk reducing breast surgery to manage cancer risk?

	1 (1)	2 (2)	3 (3)	4 (4)	5 (5)	6 (6)	7 (7)	8 (8)	9 (9)	10 (10)	
Beneficial	o	o	o	o	o	o	o	o	o	o	Harmful

Important	o	o	o	o	o	o	o	o	o	o	Unimportant
Good thing	o	o	o	o	o	o	o	o	o	o	Bad thing
Pleasant	o	o	o	o	o	o	o	o	o	o	Unpleasant

Q39 On the below, please tell us about how you feel about the option of risk reducing ovarian surgery to manage cancer risk?

	1 (1)	2 (2)	3 (3)	4 (4)	5 (5)	6 (6)	7 (7)	8 (8)	9 (9)	10 (10)	
Beneficial	o	o	o	o	o	o	o	o	o	o	Harmful
Important	o	o	o	o	o	o	o	o	o	o	Unimportant
Good thing	o	o	o	o	o	o	o	o	o	o	Bad thing
Pleasant	o	o	o	o	o	o	o	o	o	o	Unpleasant

Q40 What factors or influences have contributed to your answer above about risk management options?

____

Q41 What is your current weight? (Please specify unit of measurement)

____

Q42 Do you drink alcohol? If so, how many units on average do you drink?

	How many units?	How often?		
	Number (1)	Daily (1)	Weekly (2)	Monthly (3)
Glass of Wine (175 ml) (13)		o	o	o
Pint of Beer / Lager / Cider (568 ml) (14)		o	o	o

Bottle of Beer (Standard 330 ml) (15)		o	o	o
Alcoholic Pop Drink (275 ml) (16)		o	o	o
Shot of spirits (25 ml) includes gin, rum, vodka, whisky, tequila, sambuca (17)		o	o	o

Q43 Since having genetic testing, have you made any lifestyle changes because of your genetic test result? (i.e. stopping smoking, changing diet, etc)

____

We thank you for your time spent taking this survey, it is greatly appreciated. Your response has been recorded.

If you have any questions or concerns, please get in touch with us via the below contact details.

Cambridge - Contact us via email (cuh.precision-hboc@nhs.net), or phone, 01223 216446. Please note that this number will direct you to our main Clinical Genetics service, where you can leave a message and request for the study team to call you back.

Manchester - [Email & telephone number]

Stanford - Please direct questions to study coordinator Rozelle Laquindanum via email - rlaquind@stanford.edu, or by phone [telephone number]
